# Macrophage Migration Inhibitory Factor is subjected to glucose modification and oxidation in Alzheimer’s Disease

**DOI:** 10.1038/srep42874

**Published:** 2017-02-23

**Authors:** Omar Kassaar, Marta Pereira Morais, Suying Xu, Emily L. Adam, Rosemary C. Chamberlain, Bryony Jenkins, Tony James, Paul T. Francis, Stephen Ward, Robert J. Williams, Jean van den Elsen

**Affiliations:** 1 Department of Biology and Biochemistry, University of Bath, Bath, BA2 7AY, U.K; 2 Department of Chemistry, University of Bath, Bath, BA2 7AY, U.K; 3 Institute of Psychiatry, Psychology & Neuroscience, Wolfson Centre for Age Related Diseases, King’s College London, London, SE1 1UL, U.K; 4 Department of Pharmacy and Pharmacology, University of Bath, Bath, BA2 7AY, U.K

## Abstract

Glucose and glucose metabolites are able to adversely modify proteins through a non-enzymatic reaction called glycation, which is associated with the pathology of Alzheimer’s Disease (AD) and is a characteristic of the hyperglycaemia induced by diabetes. However, the precise protein glycation profile that characterises AD is poorly defined and the molecular link between hyperglycaemia and AD is unknown. In this study, we define an early glycation profile of human brain using fluorescent phenylboronate gel electrophoresis and identify early glycation and oxidation of macrophage migration inhibitory factor (MIF) in AD brain. This modification inhibits MIF enzyme activity and ability to stimulate glial cells. MIF is involved in immune response and insulin regulation, hyperglycaemia, oxidative stress and glycation are all implicated in AD. Our study indicates that glucose modified and oxidised MIF could be a molecular link between hyperglycaemia and the dysregulation of the innate immune system in AD.

Alzheimer’s Disease (AD) is the progressive degeneration of neurons ultimately leading to severe cognitive decline. The well characterised pathophysiology includes the presence of two hallmark proteins, amyloid-β (Aβ)^1^,^2^ and tau^3^,^4^, aggregated into insoluble plaques and neurofibrillary tangles. Several chronic metabolic states are associated with an increased incidence of AD. These include hyperglycaemia (as in diabetes), glycation^5^,^6^ and oxidative stress^7^,^8^ which could provide clues in elucidating a mechanism for AD etiology. However, the molecular link between glucose and AD are yet to be understood.

The prevalence of AD is increased in individuals with diabetes^9^, additionally, even in individuals without diabetes, higher glucose levels have been associated with an increased incidence of AD^10^. These findings suggest that hyperglycaemia, or any glucose dysregulation, could be a risk factor for AD. Accordingly, any factor involved in glucose homeostasis or insulin regulation may play a role in linking these diseases at the molecular level. Glucose, and the metabolites of glycolysis, are able to react directly with important cellular components such as DNA, lipids and protein molecules via a process known as glycation.

The process of glycation involves reducing sugar molecules such as glucose reacting with the amino groups of lysine, arginine or N-terminal amino acid residues of proteins; ultimately leading to the formation of complex and stable advanced glycation endproducts (AGEs). AGE-related modifications are present in the earliest stages of AD pathology and are thought to be involved in the formation of the pathological lesions (neurofibrillary tangles and senile plaques)^5^,^6^, since protein cross-linking is one of the effects of the AGE modification^11^. The presence of AGEs can also induce oxidative stress, either directly through their chemical development from initial glycation to end products or through interaction with cells via the AGE receptor (RAGE)^12^.

Traditionally, glycation has been detected by mass spectrometry or by using anti-AGE antibodies. More recently, a novel technique termed fluorescent phenylboronate gel electrophoresis (Flu-PAGE) has been developed^13^. Flu-PAGE exploits the reversible covalent interaction between boronic acid and *cis*-diols, that are present in fructosamine-protein adducts in glycated proteins, strengthened by the additional charge interaction between the boronate and the fructosylysine aminogroup^14^. This allows for the specific identification of glycated proteins over glycosylated and unmodified proteins in complex samples such as plasma and brain homogenates^13^. In *N*- and *O*-glycosylation these essential anomeric *cis* diols are absent precluding their interaction with the phenylboronate ligand. This highly sensitive technique detects the earliest stages of glycation, before AGEs are developed, and thus has been proposed as a tool for detecting glycated biomarkers in diseases where glycation is observed to be increased, such as diabetes^15^ and AD^5^,^6^. Our aim was to determine a glycation profile of soluble brain proteins, and to examine this glycation signature with respect to AD. Being able to assign a glycation signature specific to AD could have potential implications regarding mechanistic, diagnostic and prognostic approaches to study AD.

Here, we describe a brain glycation profile and identify macrophage migration inhibitory factor (MIF), an immune regulator and insulin regulator, as being glycated and oxidised in AD brain homogenates. The glycation completely inhibited the oxidoreductase activity of MIF, and severely attenuated its tautomerase activity. Glycation was also detrimental to the signalling effects of MIF on glia, strongly attenuating MIF-induced ERK phosphorylation, relative to unmodified MIF. These findings implicate MIF as a specific target of the precursory glycative and oxidative events in AD; providing a novel mechanistic link between diabetes and dementia.

## Results

### Glycation signature in AD brain

**Figure 1:**
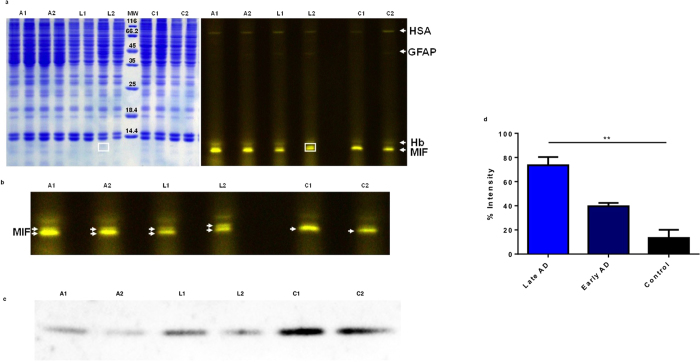
Glycation in AD brain. (**a**) Representative coomassie stained SDS-PAGE (left) and Flu-PAGE analysis (right) of brain tissue lysates from the temporal cortex of severe/late AD (A1 and A2, Braak stages V-VI), mild/early AD (L1 and L2, Braak stage II) compared with age matched controls (C1, no Braak classification and C2, Braak stage I). Glycated proteins were identified as human serum albumin (HSA), glial fibrillary acid protein (GFAP), haemoglobin (Hb) and macrophage migration inhibitory factor (MIF). Molecular weight (MW) is given in kDa (**b**) Cropped and enlarged section of the gel shown in (**a**) highlighting the difference in MIF migratory behaviour in the AD samples, compared with controls. (**c**) Western blot analysis of brain tissue lysates using anti-MIF antibody (Abcam ab55445). A Full-length gel image of this blot is shown in Supplementary Figure S11. (**d**) Quantification of MIF fluorescence intensity, presented as a percentage relative to the weakest control signal. Early AD samples (Braak stage II) show an increase in fluorescence intensity of 20%, while late AD samples (Braak stages V-VI) show an increase just over 50% relative to controls. Errors are represented as s.e.m. with n = 3. Student’s *t*-test, **P < 0.0025.

In order to assess an Alzheimer’s disease specific glycated protein signature of human brains, homogenised temporal cortex samples from severe/late AD (10 samples, Braak stages V-VI), mild/early AD (10 samples, Braak stages II-IV), and age-matched control brains (6 samples with no Braak classification, 4 samples ranging from Braak I to III) were subjected to Flu-PAGE analysis. A full definition of the Braak stage classification of each sample is provided in Supplementary Table 1. In total, ten samples of each were analysed for glycation using Flu-PAGE and subsequent MALDI-Tof MSMS mass spectrometry analysis revealed several glycated proteins including human serum albumin (HSA), glial fibrillary acid protein (GFAP), glutathione transferase (GST), haemoglobin (Hb), and MIF (Fig. 1), as summarised in Table 1.

The most striking difference between the disease and control samples was observed with MIF, appearing as a doublet in AD rather than as a single band as seen in the control samples (Fig. 1b). Although MIF is glycated in the control samples, the clear difference in the migratory behaviour of MIF identified from mild as well as severe AD samples indicate a modification specific to AD which first occurs during the early stages (Braak II) of the disease. In addition, we observe a reduction of MIF levels in both mild and severe AD samples (by 56% and 64% respectively), in comparison with age-matched controls (Fig. 1c). Conversely, quantification of the relative fluorescence intensity of glycated MIF showed an increase of 20% in early AD samples compared with controls. Significantly, the late AD samples show a 53% increase in intensity relative to controls (Fig. 1d). This analysis indicates increasing glycation of MIF as AD progresses. Since the Flu-PAGE technology takes advantage of the inherent sensitivity of fluorescence, we were able to detect far beyond the limits of conventional SDS-PAGE staining procedures, as highlighted by the white box in Fig. 1a. The absence of MIF-AGE adducts in anti-AGE and anti-carboxy-methyl lysine (anti-CML) western blot analyses of the same brain homogenate samples (Supplementary Figure S1A and S1B) indicate that the MIF carbohydrate adducts detected by Flu-PAGE comprise of *cis*-diol containing early glycation modifications. No significant differences in HSA fluorescence intensity could be observed between disease and control samples. Because of the interference by neighbouring fluorescent bands and background fluorescence, no quantitative analysis of the relative fluorescence intensity of the GFAP, GST and Hb bands could be obtained.

### MIF is glycated and oxidised in AD brain

**Figure 2:**
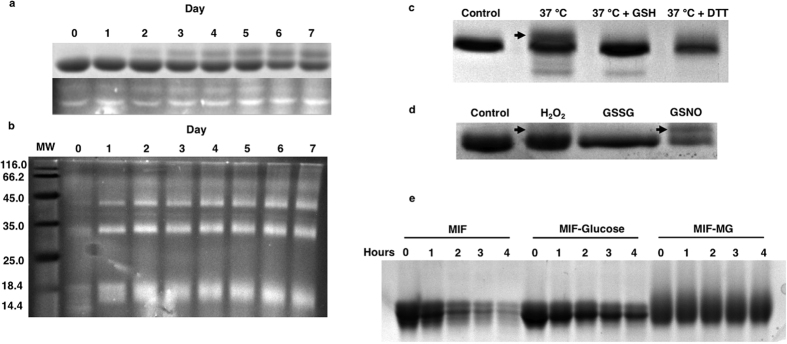
Glycation and oxidation of MIF. (**a**) Coomasie stained SDS-PAGE (top) and Flu-PAGE (bottom) analysis of recombinant MIF after glycation with glucose (50 mM) at 37 °C for 7 days. The MIF doublet can be observed developing over the time course, and is seen much earlier by Flu-PAGE. (**b**) Flu-PAGE analysis of recombinant MIF after glycating with methylglyoxal (50 mM) at 37 °C for 7 days. The MIF doublet was not reproducible using methylglyoxal, and can be seen to aggressively induce protein cross-linking. Molecular weight (MW) is given in kDa (**c**) MIF incubated in PBS alone, or in the presence of GSH (10 mM) or DTT (5 mM) at 37 °C for 4 days. Co-incubation of MIF with an antioxidant prevented or slowed the formation of the MIF doublet. (**d**) Oxidation of MIF by incubating in the presence of H_2_O_2_ (1 mM), GSSG (10 mM) or GSNO (400 μM) at 37 °C for 1 hour. The MIF doublet could be mimicked by H_2_O_2_ and GSNO under the conditions described. (**e**) Trypsin digest of unmodified and glycated MIF with glucose or MG, followed over 4 hours at 37 °C. Under these conditions, glycation was able to reduce or inhibit proteolysis by trypsin. Full-length gel images for Fig. 2(a), (c), (d) and (e) are included in Supplementary Figures S7–S9.

Following the discovery of a modified, glycated MIF in AD brain, we recombinantly expressed and purified MIF to characterise the effect of glycation on MIF activity. *In vitro* glycation reactions of MIF were conducted with glucose and followed over the course of one week using SDS-PAGE and Flu-PAGE (Fig. 2a). Control experiments were conducted in parallel where glucose was excluded from the reaction. Flu-PAGE analysis show a steadily increase of glycation over the 7-day period. Similar to the AD brain homogenates (Fig. 1a and b) we observed the formation of a MIF doublet in both SDS-PAGE and Flu-PAGE as the reaction progressed. Due to the sensitivity of fluorescent phenyl boronate, the glycated MIF doublet was first observed in the Flu-PAGE, showing an increase over time until after day 5 when the top band appeared more diffuse and less defined (Fig. 2a). Since Flu-PAGE is specific for early stage glycation, the diffuse appearance of the band may represent the development of AGE-adducts that lack *cis*-diols, thereby precluding or hindering binding of the Flu-PAGE dye. Although an increase of Flu-PAGE fluorescence was absent in the control experiment, the formation of the doublet can be seen in both samples, suggesting that an additional process is involved in this modification of MIF (Supplementary Figure S2).

In an attempt to determine the specific glycating agent causing the MIF doublet in the AD brains, a similar glycation experiment was conducted using methylglyoxal (Fig. 2b). Whilst glucose is the main energy source of the brain, methylglyoxal is an important metabolite of glycolysis and itself a significantly more reactive glycation agent *in vivo*^16^. As shown in Fig. 2b, methylglyoxal was able to rapidly glycate MIF, almost immediately inducing MIF cross-linking to form thermally and SDS resistant dimers and trimers of MIF that are visible in Flu-PAGE. Significantly, the well-defined MIF doublet observed in the AD brain samples (Fig. 1) and in the *in vitro* glycation experiments with glucose (Fig. 2a) was not observed with methylglyoxal, indicating that glucose may be responsible for the glycated MIF signature observed in the AD brains.

Although glycated MIF migrates as a doublet during gel electrophoresis, MS analysis of a glycated MIF sample revealed only a single species of the protein (Supplementary Figure S3). Glycation and oxidation are synergistic reactions and since the latter can affect the migration pattern of proteins, we repeated the MIF incubation experiments in the presence of the antioxidants glutathione and DTT in order to elucidate the underlying cause of MIF’s migratory pattern. As shown in Fig. 2c, the presence of antioxidants significantly reduced the production of the MIF doublet by 74% and 33% respectively (Fig. 2c, lane 3 and 4). To further confirm that this migratory effect is caused by the oxidation of MIF, fresh purified MIF was oxidised *in vitro* with H_2_O_2_, *S*-nitrosoglutathione (GSNO) and oxidised glutathione (GSSG) for 1 hour at 37 °C (Fig. 2d). Under these conditions, both H_2_O_2_ and GSNO were able to replicate the doublet on SDS-PAGE; thus, we were able to mimic the MIF signature observed in AD brains under these glycating and oxidising conditions.

### Glycation reduces protease susceptibility of MIF

Trypsin is the most important protease in the brain and recognises glycation targets arginine and lysine as its substrates. Whilst incubation with trypsin led to a significant degradation of the MIF control, degradation of glucose glycated MIF was greatly reduced (Fig. 2e). Because the MIF control sample can become oxidised under these test conditions, its susceptibility to trypsin indicates that this modification does not affect arginine or lysine residues in the protein. The most likely oxidation targets are MIF’s free thiols, exposed in a conserved cysteine sequence motif Cys57-Ala-Leu-Cys60 (CALC, Supplementary Figure S4), which are crucial for its enzymatic activity. The effects of glycation and oxidation on MIF function are investigated below.

### Glycation affects MIF enzymatic function

**Figure 3:**
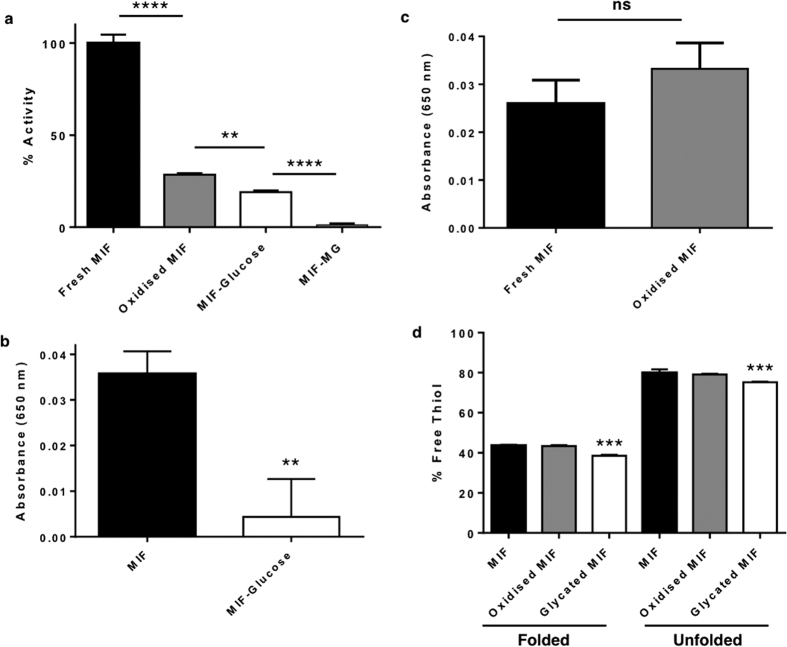
MIF enzyme activity affected by glycation and oxidation. (**a**) Tautomerase activity of MIF measured by the tautomerisation of *L*-dopachrome methyl ester at 450 nm. Data represents activity relative to unmodified MIF. Oxidation and glycation of MIF are observed to have a significant negative effect on tautomerase activity, with methylglyoxal (MG) completely inhibiting the catalysis. Errors are represented as s.e.m. with n = 7 or 8. Student’s *t*-test ****P = < 0.0001, **P = 0.0012. (**b**) Insulin reduction assay, following the MIF catalysed precipitation of the insulin b chain at 650 nm. Glycated MIF was unable to catalyse the reduction of insulin by GSH, while (**c**) *in vitro* oxidised MIF showed no significant (ns) difference in activity relative to unmodified MIF. Plot shows the reaction endpoint, with s.e.m. and n = 11. Student’s *t*-test **P = 0.0036. (**d**) Ellman’s test to determine the free thiol content of MIF under native and denaturing conditions. Oxidation under these conditions did not affect the free thiol content, and glycation showed a small reduction in available thiols. Errors expressed as s.e.m., n = 3. Student’s *t*-test ***P < 0.0003. Glycated MIF was produced by incubation at 37 °C with glucose, oxidised MIF was produced by incubation at 37 °C without glucose, as described in text. Raw tautomerase activity and insulin reduction assays are shown in Supplementary Figure S5.

MIF is part of the tautomerase superfamily^17^, and this function has been used to assess MIF activity. We utilised a tautomerase activity assay by measuring the reduction of red L-dopachrome methyl ester to colourless 5,6-dihydroxyindole-2-carboxylic acid (DHICA) at 450 nm^18^ comparing the activities of glycated MIF (incubated at 37 °C for 7 days with glucose), oxidised MIF (incubated at 37 °C for 7 days without glucose) and fresh MIF with respect to each other (Fig. 3a). Glycation with glucose was shown to significantly decrease MIF activity by 80% (p < 0.0001), while methylglyoxal glycation was strong enough to completely inhibit the tautomerase activity. Modifying MIF by oxidation alone was also shown to strongly decrease MIF tautomerase activity by 70%.

In addition to its tautomerase ability, MIF demonstrates oxidoreductase activity via its CALC motif^19^. This redox activity was analysed by an insulin reduction assay, which follows the precipitation of the insulin beta chain at 650 nm^20^. Glucose glycation of MIF completely inhibits the redox activity of the protein (p = 0.0033), with the enzyme evolving precipitation no higher than baseline (Fig. 3b). Interestingly, there was no difference in the redox activity between fresh and oxidised MIF (Fig. 3c), indicating that the oxidation of the MIF molecule occurs away from the CALC active site. The aggressive nature of cross-linking induced by methylglyoxal precluded its inclusion in these assays, due to the precipitation of MIF.

The studies into both types of MIF activity clearly show the damage caused by these modifications on the protein, with glycation shown to have more wide and salient effects. To elucidate this further, experiments to determine the free thiol content of MIF were conducted (Fig. 3d). Since thiol groups are prone to oxidation, and MIF contains three free thiols (two of which for the redox active CALC motif), a change in the free thiol content between fresh, oxidised and glycated MIF could provide clues to as to which modification is causing which effect. Free thiol content was determined using Ellman’s reagent (Ellman, 1959)^21^. These tests showed that under native conditions, there was no statistical difference between the free thiol content of fresh MIF or MIF oxidised *in vitro*. However, there was a small yet significant decrease of 4.8% (p = 0.0003) between glycated MIF compared to both fresh and oxidised MIF. Under denaturing conditions, the same trend was observed but the difference in free thiol was lower (4%). These observations show that oxidation has no direct or allosteric effect on any of the MIF free cysteines. Although a small percentage of the glycation of MIF likely occurs very close to the redox CALC site, the majority of the glycation occurs across the molecule, affecting the CALC site allosterically and inhibiting the redox activity.

### Glycation affects MIF induced ERK phosphorylation in glia

**Figure 4:**
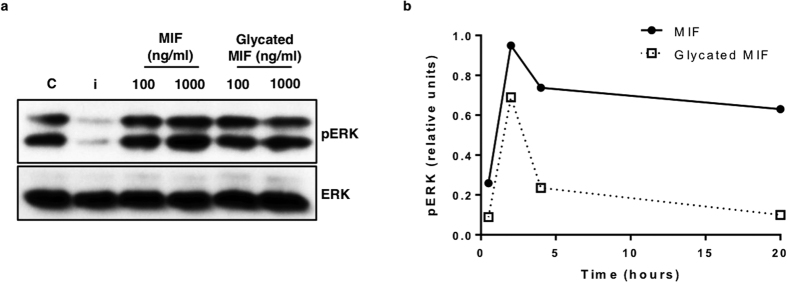
Effect of MIF glycation in primary mouse glial co-cultures of astrocytes and microglia. Cells were treated with MIF or glycated MIF (100 ng/ml or 1,000 ng/ml) and incubated at 37 °C for between 20 minutes and 20 hours. (**a**) Phosphorylation of ERK was assayed by Western blotting, with representative blots after 20 hours presented. c = PBS control, i = U0126 inhibitor, 10 μM (**b**) ERK phosphorylation at 20 minutes, 2, 4 and 20 hours. MIF was able to induce ERK phosphorylation over the full course of the experiment. Glycated MIF was still active, with attenuated activity, but was not active by the end of the 20 hour experiment. Full-length western blot images for Fig. 4(a) are included in Supplementary Figure S10.

Since MIF is known to have cytokine like activity and is involved in the regulation of both innate and adaptive immune response^22^ we investigated the effect of modified MIF on an astrocyte-microglia cell coculture. The cells were treated with fresh MIF or glycated MIF at 100 or 1,000 ng/ml. The effect of MIF on microglia activation and signalling was determined by measuring the levels of phosphorylated ERK1/2 (Fig. 4a). ERK phosphorylation was increased by almost 70% at 20 hours upon treatment with MIF (1,000 ng/ml), but remained at baseline when treated with glycated MIF. When the ERK activation was followed for 20 hours, glycation of MIF was shown to significantly attenuate its activity, but was not shown to be completely inactive. In line with the tautomerase activity assay results, glycated MIF was still active to a degree, being able to promote ERK phosphorylation after 2 and 4 hours. However, the ERK phosphorylation was returned to baseline by 20 hours, while fresh, unmodified MIF was still showing significant activity (Fig. 4b).

## Discussion

Glycation and oxidation are both prominent and recurring features of AD pathology. AGE development is accelerated by reactive oxygen species and involves oxidation reactions; while in parallel, generates superoxide radicals and can trigger oxidative stress through AGE-RAGE interactions and microglia activation^12^. Hyperglycaemia, which leads to increased glycation, is also associated with AD; but a molecular link remains to be determined.

Using Flu-PAGE to determine a glycation signature of human brain, we analysed AD and age-matched control brain samples and found HSA, GFAP, GST, Hb (β subunit) and MIF to be glycated in AD and age-matched control brain samples (Table 1). HSA and Hb are among the most sensitive proteins for modification by glucose. Glycated HSA and Hb are abundant in Diabetes sufferers and can be used to assess glycemic control and to modify therapy. Glycated GFAP has been linked to AD before with the observation of intracellular AGE deposits co-localising with GFAP-positive astrocytes in AD brain^23^. Apart from MIF, no striking differential glycation profiles have been observed for these proteins between AD and control brain lysates. However, other, yet to be identified proteins show clearly distinct Flu-PAGE patterns, as illustrated by Supplementary Figure S6. Some of these include a significant number of proteins that fall within the molecular weight range expected of tau as observed by anti-tau western blot (Supplementary Figure S6B). Anti-AGE and anti-CML (Supplementary Figure S1A and B) suggest that protein bands in the tau protein molecular weight range are indeed modified with advanced glycation end products, consistent with the literature^24^. These proteins and others will be explored in future studies. In comparison with our previous study on a double transgenic AD mouse model^13^, TASTPM, only the detection of glycated GST correlates with the results from this human study although no significant changes in GST glycation patterns have yet been detected in the human brain samples. The TASTPM mouse model contains the Swedish mutation in APP and the M146V mutation in PSEN1, and thus develops the amyloidogenic pathology of AD, indicating that the glycation signature observed in human brains is probably not driven by APP processing but rather by other molecular mechanisms, such as oxidative stress.

Specifically, we identified significant differences in the glycation and migration profile of MIF between AD brain samples and age-matched controls. These differences appear to involve early carbohydrate modifications, as indicated by the absence of AGE-modified MIF in anti-AGE and anti-CML western blot analyses of the brain tissue lysates (Supplementary Figure S1A and S1B). MIF is an important immune regulatory molecule with cytokine-like pro-inflammatory properties, which is upregulated in conditions of oxidative stress and chronic inflammation^25^,^26^. MIF is known to bind Aβ^27^, activate microglia via the receptor CD74^28^,^29^,^30^ and has been observed in the cerebrospinal fluid of AD patients at significantly higher levels relative to controls^31^. MIF is also involved in insulin regulation, glucose homeostasis and diabetes^32^,^33^,^34^,^35^. There were three key outcomes from this part of the study within the wider context of AD: 1) the identification of a pro-inflammatory cytokine, MIF, which has been linked to neuroinflammation in early AD^36^, 2) MIF was detected using Flu-PAGE, which detects early glycation modifications on proteins, 3) glycation of MIF increases as AD progresses from Braak stages I to VI. Thus, an immunoregulatory and insulin regulating protein was shown to be modified in the early stages of AD progression, which is vital in developing of a chronology of the pathology in AD progression.

The most striking difference in the appearance of MIF in AD relative to controls is its migration pattern; MIF appeared as a distinct doublet with significantly increased fluorescence intensity on Flu-PAGE in the mild (Braak II-IV) and late (Braak V-VI) stage AD samples. The increased MIF fluorescence intensity observed in mild and late stage AD samples is not caused by increased levels of MIF protein in these samples, as anti-MIF western blot analysis shows the inverse result. The observation of reduced MIF levels in the disease samples, however, may result from a loss of specific binding by the anti-MIF antibodies caused by altered epitopes on the surface of MIF, as a consequence of the disease-linked glycoxidation. To determine the cause and significance of the doublet migratory pattern of MIF, *in vitro* glycation studies using recombinant MIF were conducted. These experiments showed that a glycation pattern similar to that observed in AD brain homogenates could be reproduced by incubating MIF with glucose. Methylglyoxal glycation was unable to reproduce this pattern, leading instead to almost immediate MIF-crosslinking, aggregation and diffuse bands. These observations suggest that glucose may be responsible for the glycation observed in the brain homogenates, but does not rule out the possibility of the involvement of other reducing sugars. Nevertheless, we can exclude glycation by methylglyoxal from the process identified in AD brain. This is significant since methylglyoxal is considered to be the main agent for glycation and therefore AGEs^16^. The body has evolved the capacity to regulate glycation by detoxifying glucose metabolites which form as byproducts of glycolysis^37^ (such as methylglyoxal and glyoxal), but no such system is currently known for glucose modified proteins.

The formation of a MIF doublet in the control samples of the *in vitro* glucose incubations indicate that glycation alone cannot explain the migration behaviour of MIF identified in the AD brain samples. To understand this, *in vitro* redox experiments were designed to ascertain the effect of oxidation on MIF migration through SDS-PAGE, since oxidative stress has been a recurring theme in the field of AD research. In experiments where MIF was incubated in the presence of an antioxidant (GSH or DTT), the formation of the doublet was either significantly reduced or completely abrogated. To support this further, *in vitro* oxidation of MIF was shown to produce the doublet migration pattern when oxidising with H_2_O_2_ or GSNO. Although at this moment we cannot ascribe the oxidation of MIF that occurs *in vivo* to a specific oxidising agent, the fact that GSH is able to prevent MIF oxidation *in vitro* is an important observation within the context of AD. An imbalance in the redox state of the brain, particularly the levels of GSH which are significantly decreased, is now considered to be an important parameter of the disease state^38^. We were also able to replicate the MIF doublet using GSNO, an endogenous nitric oxide donor, suggesting that reactive nitrogen species may be involved in modifying MIF in AD. A homologue of MIF via the thioredoxin family, protein disulphide isomerase, has been shown to be nitrosylated in AD^39^. More recently, high glucose induced redox-mediated modifications of proteins have been suggested as a pathway linking hyperglycaemia to cognitive decline in AD^40^. Taken all together, these data show that MIF in AD brain was modified by both glycation and oxidation, to produce the observed pattern. These salient findings link two processes (glycation and oxidation) that have previously been considered as separate pathways which may be involved in AD, to one immune and glucose regulatory molecule, MIF.

To assess the activity of the modified MIF, we used two standard MIF enzymatic assays to determine its tautomerase and oxidoreductase efficacy. Although no endogenous tautomerase substrate is currently known, inhibitors of the MIF tautomerase activity have been shown to attenuate and inhibit the immune response signal induced by MIF^41^. Glycation with glucose was shown to significantly reduce MIF tautomerase activity, while methylglyoxal completely inactivated tautomerase activity. Oxidation of MIF alone was also shown to have a significant effect; an effect which was compounded further by glycation. Another aspect of glycation is to cause cross-linking between molecules via adducts on lysine or arginine residues, in effect protecting the protein from degradation by trypsin; one of the predominant proteases spread widely across the brain^42^. This may enable MIF to persist, existing with a lowered activity, but a prolonged half-life since its clearance would be affected. MIF oxidoreductase activity was assayed for using an insulin precipitation assay, which showed glycation was able to completely inhibit the redox activity of the protein. Particularly in recent years, oxidative stress and the redox balance of the brain have been highlighted as potential triggers in the progression of AD^7^. With MIF being a redox active protein (and is part of the thioredoxin family^43^), any alteration in its redox behaviour could play a role in perturbing natural redox homeostasis. Interestingly, oxidation of MIF had no effect on its ability to catalyse the reduction of the insulin disulphide bridges. The tautomerase and oxidoreductase sites are at two different regions of the molecule, providing a structural clue for the site of the *in vitro* oxidation (since the tautomerase activity was affected, while the redox activity remained). The MIF monomer contains three free thiols, two of which are part of the redox CALC motif; an Ellman’s test to quantify the free thiol content of modified MIF was carried out. Oxidation was found to have no effect on the free thiol content, indicating that the oxidation of MIF occurs away from the CALC active site. Glycation was found to have a small, but statistically significant effect, decreasing the free thiol content by almost 5%. The fact that this was the same for both folded and unfolded MIF experiments suggests that at least some small proportion of the glycation occurs very close to (or even directly on) the cysteine residues of the CALC motif. Nevertheless, the majority of the impact on MIF redox activity is likely to come from allosteric modulation of the MIF structure from glycation around the molecule, or by reducing the flexibility of the MIF molecule. However, this test cannot rule out the possibility of the *in vivo* oxidation of MIF occurring at one of the free thiols, such as *S*-nitrosylation, as mimicked earlier by incubating MIF with GSNO.

We have shown that glucose modification and oxidation of MIF can severely affect MIF activity. Modified MIF activity would have implications for the innate immune response to any nascent pathological AD lesions. Interestingly, MIF modulates insulin secretion, is involved in glucose homeostasis and has been associated with diabetes and hyperglycaemia^32^,^33^,^34^. Chronic inflammation, a common risk factor of diabetes and obesity, induced by the proinflammitory action of MIF is associated with insulin resistance and glucose intolerance^35^. In obesity, plasma MIF concentrations are increased, but treatment with the anti-diabetic and anti-glycation agent metformin, decreases the MIF levels to normal^44^. This context provides an intriguing basis for linking hyperglycaemia to dementia through glycation and inflammation.

The natural immune response to homeostatic disruption in the central nervous system is led by microglia cells, acting as sentinels over the cellular regulation of the brain by responding to pathogens, neuronal apoptosis and other signs of damage such as protein aggregates^45^. In AD, microglia are closely associated with the disease hallmarks; soluble and fibrillary Aβ can be internalised and degraded by microglia, and pathological tau is able to strongly activate microglial response^46^. However, microglial activation is a dynamic process and questions remain as to whether microglia cells play a neuroprotective^47^,^48^,^49^ or a neurodegenerative^50^,^51^,^52^ role in AD progression. Recent reviews suggest that an aberrant microglial immune response develops in the course of the disease, switching from a beneficial to dystopic microglial mode of action, ultimately spreading AD pathology across the brain^53^. Since the enzymatic activity of MIF was shown to be severely hindered or even completely inhibited by glycation, we investigated the effect of this observation on the ability of MIF to communicate with cells.

A glial coculture of microglia and astrocytes was treated with fresh purified MIF or modified glycated MIF. These preliminary experiments looked at the phosphorylation of ERK as a signal induced by MIF over a period of 20 hours. MIF was able to induce ERK phosphorylation for up 20 hours, correlating with previous studies^54^. However, glycated MIF was unable to sustain activity, and showed an attenuated profile over the 20 hour experiment. Interestingly though, in parallel with the tautomerase activity experiments, glycated MIF was still active to some extent. These primary observations relate directly to the current trend in AD research, where microglia malfunction is being presented as the instigation from the clinically silent tau tangles and amyloid fibrils to the pathological neurodegeneration in AD^53^. If microglia are involved in internalising soluble forms of Aβ fibrils or extracellularly degrading Aβ plaques, their inability to respond in the early stages of AD could be the tipping point for disease progression. In addition, MIF has been shown to bind Aβ, and is associated around Aβ plaques with microglia; indicating perhaps a directory role for MIF in the microglial response^27^. These initial studies on the effect of glycated MIF on glial cells present many questions, which when answered, could lead to a greater understanding of the mechanism of AD.

The study presented here identified MIF in AD brain to be both glycated and oxidised. Both processes are known to occur during the course of AD, and with this new evidence, we are able to conclude that the glycation and oxidation of MIF occurs during the early stages of the disease. Glycation has a significant effect on MIF activity, and its ability to communicate with the immune system, in this case glial cells. Since MIF is one of the first modulators of immune response, is implicated in diabetes and insulin secretion, these findings provide a perspective which resonates with current AD hypotheses; glucose modified and oxidised MIF may link hyperglycaemia, glycation, oxidative stress, and impaired immune response to the cognitive decline observed in dementia.

## Conclusions

Glucose and glucose metabolites are known to adversely modify proteins through glycation, a characteristic of hyperglycaemia. This study set out to define the protein glycation profile of human brain, using fluorescent phenylboronate gel electrophoresis, with the aim to identify disease specific glucose modifications. This novel technique exploits the reversible covalent interaction between boronic acid and the cis-diols of the fructosamine-protein adduct in glycated proteins, allowing for their identification over unmodified proteins in a variety of complex samples, including plasma and brain homogenates. By applying this novel chemical tool we have been able to: 1) visualise and identify a profile of hitherto undetectable early glycation adducts in human brains that precede the formation of the advanced glycation end-products (AGEs), which are associated with AD pathology. 2) Significantly, we identified an oxidised variant of glucose-modified macrophage migration inhibitory factor (MIF) in both early and late stage AD brains. 3) We also show that these modifications can develop within a matter of days *in vitro* and can affect MIF activity and degradation long before the formation of irreversible AGEs. 4) Since MIF is a key immunoregulator and is also involved in insulin regulation, our findings implicate glucose modified and oxidised MIF as a molecular link between hyperglycaemia, oxidative stress and dysregulation of the innate immune system in AD.

## Methods

### Human AD brain samples

Tissue for this study was provided by the London Neurodegenerative Brain Bank which is funded by grants from the UK Medical Research Council and by Brains for Dementia Research, a joint venture between Alzheimer’s Society and Alzheimer’s Research UK. 10 samples were classified as severe/late AD (Braak stage V-VI). 10 were classified as mild/early AD (Braak stage II-IV). Out of 10 controls, 6 were classified as normal or with mild ageing changes and 4 were classified as normal but with modified Braak stages ranging from I-III.

### Flu-PAGE analysis

Glycated proteins were identified as previously described^13^. Briefly, brain homogenates were incubated with 0.5 mM fluorescent boronic acid at room temperature for one hour. Gel electrophoresis was performed using an Xcell surelock mini-cell (Invitrogen), separating proteins on 15% tris-glycine gels. Glycation was visualised prior to protein staining with a Dark Reader^®^ (Clare Chemicals Research Inc.; 420–520 nm, with 530 or 595 nm filter). Gels were then stained with coomassie to visualise total protein content. Samples where only SDS-PAGE was required skipped the boronic acid incubation and visualisation stages. Quantification of protein band fluorescence and coomassie stain intensity was performed using Fusion software (Vilber Lourmat).

### Identification of glycated proteins by mass spectrometry

Glycated protein bands identified by Flu-PAGE analysis were excised from the Coomassie stained gel and subjected to in-gel proteolytic trypsin digestion using an automated DigestPro digestion unit at the University of Bristol Proteomics Facility. The resulting peptides were subsequently analysed by MALDI-Tof MSMS using a Bruker Daltonics UltrafleXtreme 2 mass spectrometer. Database searches were performed using the facility’s Mascot server to identify the protein.

### Expression and purification of recombinant MIFh

His-tagged MIF was expressed in BL21 *E.coli* cells containing the pET15b-MIF vector, and purified using a HiTrap™ HP nickel column (GE) under standard conditions. Elution of MIF was achieved with 500 mM imidazole. His-tag was removed by adding 50 U/ml bovine thrombin (CalBiochem^®^ MerckMillipore) and dialysed into PBS. Thrombin was removed by using a HiTrap™ benzamidine FF (GE).

### *In vitro* glycation and oxidation of MIF

Typically, protein glycation of recombinant MIF (0.5 mg/ml) was carried out over 7 days at 37 °C, with either glucose or methylglyoxal (50 mM) in PBS. Control samples were performed under the same conditions without carbohydrate. Samples were taken each day to observe a time course of the reaction.

To assess the effect of antioxidants on MIF over time, MIF (0.5 mg/ml) was incubated in PBS alone or in the presence of the reducing agents GSH (10 mM) or DTT (5 mM) at 37 °C and the reaction proceeded over 5 days. Conversely, MIF (0.5 mg/ml) was oxidised *in vitro* by incubated in PBS alone or in the presence of the oxidising agents H_2_O_2_ (1 mM), GSSG (10 mM) or GSNO (400 μM) at 37 °C and the reaction allowed to proceed for 1 hour. A fresh frozen MIF sample was used as control. Samples were separated by SDS-PAGE and stained as above.

### Trypsin digestion of glycated MIFh

The proteolytic stability of glycated MIF was examined by trypsin digestion. Glycated MIF (0.5 mg/ml) prepared as above was incubated with trypsin at a 200:1 ratio. The solution was incubated at 37 °C for 4 hours. A control reaction with unmodified MIF was performed simultaneously. Samples were separated by SDS-PAGE and stained as above.

### MIF activity assays

MIF tautomerase activity was measured by following a modified L-Dopachrome Methyl Ester Tautomerase assay protocol^18^. Reactions contained 1.82 μM MIF, 0.57 mM NaI and 0.29 mM L-dopa in sodium phosphate buffer at pH 6.2. The decrease in absorbance at 450 nm was followed for 15 minutes. MIF oxidoreductase activity was measured using an insulin precipitation assay^20^. Briefly, the reaction mixture was comprised of 12.5 μM MIF, 78 μg/ml insulin solution, 10 mM GSH in PBS containing 2 mM EDTA at pH 7.5. Solution turbidity was measured at 650 nm for up to 2 hours.

### Free thiol content determination

MIF free thiol levels were determined using Ellman’s reagent (Ellman, 1959)^21^. MIF (0.5 mg/ml) was incubated with 5,5′-dithio-bis-(2-nitrobenzoic acid) in PBS pH 8.0, containing 1 mM EDTA and incubated at room temperature for 15 minutes. Absorbance was read at 415 nm, and a molar extinction coefficient of 14,150 M^−1^cm^−1^.

### Primary cortical glial cell culture and treatment with MIF and glycated/oxidised MIF

#### Animal Ethics Statement

Primary cortical glial cultures were prepared from mouse embryos in accordance with UK Home Office Guidelines as stated in the Animals (Scientific Procedures) Act 1986 using Schedule 1 procedures approved by the University of Bath Animal Welfare and Ethical Review Body.

#### Primary cortical glial cell culture

Primary cortical glial cells from mouse embryos we prepared essentially as previously described^55^. Briefly, cortices were dissected from embryonic day 15 CD1 mouse embryos and mechanically dissociated using a fire-polished glass Pasteur pipette in PBS (Ca^2+^- and Mg^2+^-free) supplemented with 33 mM glucose. Cells were plated into Nunc (Rochester, NY) multiwell tissue culture plates that had been coated previously with 20 μg/ml poly-D-lysine (Sigma, St. Louis, MO) and were maintained in DMEM-F12 medium, supplemented with 2 mM glutamine, 100 μg/ml streptomycin, and 60 μg/ml penicillin and 10% heat inactivated fetal bovine serum (Invitrogen, Carlsbad, CA), at 37 °C in a humidified atmosphere of 95% air and 5% CO_2_. Mixed glial cultures used after 14 days *in vitro* (DIV), were composed of ~80% astrocytes and ~15–20% microglia as judged by GFAP and CD11b staining respectively (not shown). Neuronal elements were typically less than 2%, based on β-tubulin III staining.

#### MIF treatment of primary cortical glial cells

MIF was purified and modified as described above. Cells were treated with 100 or 1,000 ng/ml of fresh purified or modified MIF and ERK phosphorylation detected over 20 hours by Western Blot, using anti-phosphoERK (Cell Signalling) and anti-ERK2 (Santa Cruz Biotechnology). Blots were visualised on film and quantified by Fusion software (Viber Lourmat).

### Statistical analysis

Statistical analysis was performed using Graphpad Prism software and data are presented as mean ± s.e.m. Students *t*-tests were used to show statistical significance for single comparisons.

## Additional Information

**How to cite this article**: Kassaar, O. *et al*. Macrophage Migration Inhibitory Factor is subjected to glucose modification and oxidation in Alzheimer’s Disease. *Sci. Rep.* **7**, 42874; doi: 10.1038/srep42874 (2017).

**Publisher's note:** Springer Nature remains neutral with regard to jurisdictional claims in published maps and institutional affiliations.

## Supplementary Material

Supplementary Information

## Figures and Tables

**Table 1:** Glycation signature of aged human brain as determined by Flu-PAGE analsysis followed by mass spectrometry identification.

Glycated Protein	Abbreviation	Molecular Weight (kDa)	Association with AD	Reference
Human serum albumin	HSA	65	Regulates Aβ fibril formation. Glycation induces cytotoxicity.	56,57
Glial fibrillary acidic protein	GFAP	45	Increased expression. Modifications linked to AD.	58, 59, 60
Glutathione transferase	GST	25	Decreased levels and activity.	13,61,62
Haemoglobin (β chain)	Hb	15	Decreased levels. Co-localised with Aβ plaques.	63,64
Macrophage migration inhibitory factor	MIF	13	Binds Aβ. Increased levels in cerebrospinal fluid.	27,31

Although the identified proteins are glycated in both AD and control brain samples they have been associated with AD in literature.

## References

[b1] Selkoe D. J. Alzheimer’s disease: genes, proteins, and therapy. Physiol. Rev. 81, 741–66 (2001). 11274343 10.1152/physrev.2001.81.2.741

[b2] Selkoe D. J. Alzheimer’s disease results from the cerebral accumulation and cytotoxicity of amyloid beta-protein. J. Alzheimers. Dis. 3, 75–80 (2001). 12214075 10.3233/jad-2001-3111

[b3] Querfurth H. W. & LaFerla F. M. Alzheimer’s Disease. N. Engl. J. Med. 362, 329–344 (2010). 20107219 10.1056/NEJMra0909142

[b4] Grundke-Iqbal I. . Abnormal phosphorylation of the microtubule-associated protein tau (tau) in Alzheimer cytoskeletal pathology. Proc. Natl. Acad. Sci. USA 83, 4913–7 (1986). 3088567 10.1073/pnas.83.13.4913 PMC323854

[b5] Vitek M. P. . Advanced glycation end products contribute to amyloidosis in Alzheimer disease. Proc. Natl. Acad. Sci. USA 91, 4766–70 (1994). 8197133 10.1073/pnas.91.11.4766 PMC43869

[b6] Smith M. A. . Advanced Maillard reaction end products are associated with Alzheimer disease pathology. Proc. Natl. Acad. Sci. USA 91, 5710–4 (1994). 8202552 10.1073/pnas.91.12.5710 PMC44066

[b7] Bonda D. J. . Neuronal failure in Alzheimer’s disease: a view through the oxidative stress looking-glass. Neurosci. Bull. 30, 243–52 (2014). 24733654 10.1007/s12264-013-1424-x PMC4097013

[b8] Rosini M., Simoni E., Milelli A., Minarini A. & Melchiorre C. Oxidative stress in Alzheimer’s disease: are we connecting the dots? J. Med. Chem. 57, 2821–31 (2014). 24131448 10.1021/jm400970m

[b9] Schrijvers E. M. C. . Insulin metabolism and the risk of Alzheimer disease: the Rotterdam Study. Neurology 75, 1982–7 (2010). 21115952 10.1212/WNL.0b013e3181ffe4f6 PMC3014236

[b10] Crane P. K. . Glucose levels and risk of dementia. N. Engl. J. Med. 369, 540–8 (2013). 23924004 10.1056/NEJMoa1215740 PMC3955123

[b11] Eble A. S., Thorpe S. R. & Baynes J. W. Nonenzymatic glucosylation and glucose-dependent cross-linking of protein. J. Biol. Chem. 258, 9406–12 (1983). 6409904

[b12] Yan S. D. . RAGE and amyloid-beta peptide neurotoxicity in Alzheimer’s disease. Nature 382, 685–91 (1996). 8751438 10.1038/382685a0

[b13] Pereira Morais M. P. . Analysis of protein glycation using fluorescent phenylboronate gel electrophoresis. Sci. Rep. 3, 1437 (2013). 23531746 10.1038/srep01437 PMC3609018

[b14] Morais M. P. P. . Analysis of protein glycation using phenylboronate acrylamide gel electrophoresis. Proteomics 10, 48–58 (2010). 19899078 10.1002/pmic.200900269

[b15] Unoki H. & Yamagishi S. Advanced glycation end products and insulin resistance. Curr. Pharm. Des. 14, 987–9 (2008). 18473850 10.2174/138161208784139747

[b16] Rabbani N. & Thornalley P. J. Measurement of methylglyoxal by stable isotopic dilution analysis LC-MS/MS with corroborative prediction in physiological samples. Nat. Protoc. 9, 1969–79 (2014). 25058644 10.1038/nprot.2014.129

[b17] Poelarends G. J., Veetil V. P. & Whitman C. P. The chemical versatility of the beta-alpha-beta fold: catalytic promiscuity and divergent evolution in the tautomerase superfamily. Cell. Mol. Life Sci. 65, 3606–18 (2008). 18695941 10.1007/s00018-008-8285-x PMC2930816

[b18] Healy Z. R., Liu H., Holtzclaw W. D. & Talalay P. Inactivation of tautomerase activity of macrophage migration inhibitory factor by sulforaphane: a potential biomarker for anti-inflammatory intervention. Cancer Epidemiol. Biomarkers Prev. 20, 1516–23 (2011). 21602309 10.1158/1055-9965.EPI-11-0279 PMC3132381

[b19] Kleemann R. . Disulfide analysis reveals a role for macrophage migration inhibitory factor (MIF) as thiol-protein oxidoreductase. J. Mol. Biol. 280, 85–102 (1998). 9653033 10.1006/jmbi.1998.1864

[b20] Kleemann R., Mischke R., Kapurniotu A., Brunner H. & Bernhagen J. Specific reduction of insulin disulfides by macrophage migration inhibitory factor (MIF) with glutathione and dihydrolipoamide: potential role in cellular redox processes. FEBS Lett. 430, 191–6 (1998). 9688536 10.1016/s0014-5793(98)00654-1

[b21] Ellman G. L. Tissue sulfhydryl groups. Arch. Biochem. Biophys. 82, 70–7 (1959). 13650640 10.1016/0003-9861(59)90090-6

[b22] Calandra T. & Roger T. Macrophage migration inhibitory factor: a regulator of innate immunity. Nat. Rev. Immunol. 3, 791–800 (2003). 14502271 10.1038/nri1200 PMC7097468

[b23] Horie K. . Immunohistochemical Localization of Advanced Glycation End Products, Pentosidine, and Carboxymethyllysine in Lipofuscin Pigments of Alzheimer’s Disease and Aged Neurons. Biochem. Biophys. Res. Commun. 236, 327–332 (1997). 9240434 10.1006/bbrc.1997.6944

[b24] Yan S. D. . Non-enzymatically glycated tau in Alzheimer’s disease induces neuronal oxidant stress resulting in cytokine gene expression and release of amyloid beta-peptide. Nat. Med. 1, 693–9 (1995). 7585153 10.1038/nm0795-693

[b25] Baugh J. A. & Bucala R. Macrophage migration inhibitory factor. Crit. Care Med. 30, S27–35 (2002). 11782558

[b26] Grieb G., Merk M., Bernhagen J. & Bucala R. Macrophage migration inhibitory factor (MIF): a promising biomarker. Drug News Perspect. 23, 257–64 (2010). 20520854 10.1358/dnp.2010.23.4.1453629 PMC3131110

[b27] Oyama R., Yamamoto H. & Titani K. Glutamine synthetase, hemoglobin alpha-chain, and macrophage migration inhibitory factor binding to amyloid beta-protein: their identification in rat brain by a novel affinity chromatography and in Alzheimer’s disease brain by immunoprecipitation. Biochim. Biophys. Acta 1479, 91–102 (2000). 11004532 10.1016/s0167-4838(00)00057-1

[b28] Zeiner P. S. . MIF Receptor CD74 is Restricted to Microglia/Macrophages, Associated with a M1-Polarized Immune Milieu and Prolonged Patient Survival in Gliomas. Brain Pathol. 25, 491–504 (2015). 25175718 10.1111/bpa.12194 PMC8029437

[b29] Cox G. M. . Macrophage migration inhibitory factor potentiates autoimmune-mediated neuroinflammation. J. Immunol. 191, 1043–54 (2013). 23797673 10.4049/jimmunol.1200485

[b30] Wang F. . Macrophage migration inhibitory factor activates cyclooxygenase 2-prostaglandin E2 in cultured spinal microglia. Neurosci. Res. 71, 210–8 (2011). 21802455 10.1016/j.neures.2011.07.1821

[b31] Bacher M. . The role of macrophage migration inhibitory factor in Alzheimer’s disease. Mol. Med. 16, 116–21 (2010). 20200619 10.2119/molmed.2009.00123 PMC2829616

[b32] Vujicic M. . The critical role of macrophage migration inhibitory factor in insulin activity. Cytokine 69, 39–46 (2014). 25022960 10.1016/j.cyto.2014.05.013

[b33] Morrison M. C. & Kleemann R. Role of Macrophage Migration Inhibitory Factor in Obesity, Insulin Resistance, Type 2 Diabetes, and Associated Hepatic Co-Morbidities: A Comprehensive Review of Human and Rodent Studies. Front. Immunol. 6, 308 (2015). 26124760 10.3389/fimmu.2015.00308 PMC4467247

[b34] Stosic-Grujicic S. . Macrophage migration inhibitory factor (MIF) is necessary for progression of autoimmune diabetes mellitus. J. Cell. Physiol. 215, 665–75 (2008). 18064633 10.1002/jcp.21346

[b35] Verschuren L. . MIF deficiency reduces chronic inflammation in white adipose tissue and impairs the development of insulin resistance, glucose intolerance, and associated atherosclerotic disease. Circ. Res. 105, 99–107 (2009). 19478200 10.1161/CIRCRESAHA.109.199166 PMC2717797

[b36] Popp J. . Macrophage migration inhibitory factor in mild cognitive impairment and Alzheimer’s disease. J. Psychiatr. Res. 43, 749–53 (2009). 19038405 10.1016/j.jpsychires.2008.10.006

[b37] Maessen D. E. M., Stehouwer C. D. A. & Schalkwijk C. G. The role of methylglyoxal and the glyoxalase system in diabetes and other age-related diseases. Clin. Sci. (Lond). 128, 839–61 (2015). 25818485 10.1042/CS20140683

[b38] Mandal P. K., Saharan S., Tripathi M. & Murari G. Brain glutathione levels–a novel biomarker for mild cognitive impairment and Alzheimer’s disease. Biol. Psychiatry 78, 702–10 (2015). 26003861 10.1016/j.biopsych.2015.04.005

[b39] Uehara T. . S-nitrosylated protein-disulphide isomerase links protein misfolding to neurodegeneration. Nature 441, 513–7 (2006). 16724068 10.1038/nature04782

[b40] Akhtar M. W. . Elevated glucose and oligomeric β-amyloid disrupt synapses via a common pathway of aberrant protein S-nitrosylation. Nat. Commun. 7, 10242 (2016). 26743041 10.1038/ncomms10242 PMC4729876

[b41] Pantouris G. . An Analysis of MIF Structural Features that Control Functional Activation of CD74. Chem. Biol. 22, 1197–205 (2015). 26364929 10.1016/j.chembiol.2015.08.006 PMC4575896

[b42] Wang Y., Luo W. & Reiser G. Trypsin and trypsin-like proteases in the brain: proteolysis and cellular functions. Cell. Mol. Life Sci. 65, 237–52 (2008). 17965832 10.1007/s00018-007-7288-3 PMC11131809

[b43] Son A. . Direct association of thioredoxin-1 (TRX) with macrophage migration inhibitory factor (MIF): regulatory role of TRX on MIF internalization and signaling. Antioxid. Redox Signal. 11, 2595–605 (2009). 19601712 10.1089/ars.2009.2522

[b44] Dandona P. . Increased plasma concentration of macrophage migration inhibitory factor (MIF) and MIF mRNA in mononuclear cells in the obese and the suppressive action of metformin. J. Clin. Endocrinol. Metab. 89, 5043–7 (2004). 15472203 10.1210/jc.2004-0436

[b45] Prinz M. & Priller J. Microglia and brain macrophages in the molecular age: from origin to neuropsychiatric disease. Nat. Rev. Neurosci. 15, 300–12 (2014). 24713688 10.1038/nrn3722

[b46] Prokop S., Miller K. R. & Heppner F. L. Microglia actions in Alzheimer’s disease. Acta Neuropathol. 126, 461–77 (2013). 24224195 10.1007/s00401-013-1182-x

[b47] Condello C., Yuan P., Schain A. & Grutzendler J. Microglia constitute a barrier that prevents neurotoxic protofibrillar Aβ42 hotspots around plaques. Nat. Commun. 6, 6176 (2015). 25630253 10.1038/ncomms7176 PMC4311408

[b48] Paresce D. M., Ghosh R. N. & Maxfield F. R. Microglial cells internalize aggregates of the Alzheimer’s disease amyloid beta-protein via a scavenger receptor. Neuron 17, 553–65 (1996). 8816718 10.1016/s0896-6273(00)80187-7

[b49] Mandrekar S. . Microglia mediate the clearance of soluble Abeta through fluid phase macropinocytosis. J. Neurosci. 29, 4252–62 (2009). 19339619 10.1523/JNEUROSCI.5572-08.2009 PMC3034143

[b50] McDonald D. R., Brunden K. R. & Landreth G. E. Amyloid fibrils activate tyrosine kinase-dependent signaling and superoxide production in microglia. J. Neurosci. 17, 2284–94 (1997). 9065490 10.1523/JNEUROSCI.17-07-02284.1997 PMC6573513

[b51] Combs C. K., Johnson D. E., Karlo J. C., Cannady S. B. & Landreth G. E. Inflammatory mechanisms in Alzheimer’s disease: inhibition of beta-amyloid-stimulated proinflammatory responses and neurotoxicity by PPARgamma agonists. J. Neurosci. 20, 558–67 (2000). 10632585 10.1523/JNEUROSCI.20-02-00558.2000 PMC6772401

[b52] Combs C. K., Johnson D. E., Cannady S. B., Lehman T. M. & Landreth G. E. Identification of microglial signal transduction pathways mediating a neurotoxic response to amyloidogenic fragments of beta-amyloid and prion proteins. J. Neurosci. 19, 928–39 (1999). 9920656 10.1523/JNEUROSCI.19-03-00928.1999 PMC6782151

[b53] Mhatre S. D., Tsai C. A., Rubin A. J., James M. L. & Andreasson K. I. Microglial malfunction: the third rail in the development of Alzheimer’s disease. Trends Neurosci. 38, 621–36 (2015). 26442696 10.1016/j.tins.2015.08.006 PMC4670239

[b54] Mitchell R. A., Metz C. N., Peng T. & Bucala R. Sustained mitogen-activated protein kinase (MAPK) and cytoplasmic phospholipase A2 activation by macrophage migration inhibitory factor (MIF). Regulatory role in cell proliferation and glucocorticoid action. J. Biol. Chem. 274, 18100–6 (1999). 10364264 10.1074/jbc.274.25.18100

[b55] Bahia P. K., Rattray M. & Williams R. J. Dietary flavonoid (-)epicatechin stimulates phosphatidylinositol 3-kinase-dependent anti-oxidant response element activity and up-regulates glutathione in cortical astrocytes. J. Neurochem. 106, 2194–204 (2008). 18624917 10.1111/j.1471-4159.2008.05542.x

[b56] Stanyon H. F. & Viles J. H. Human serum albumin can regulate amyloid-β peptide fiber growth in the brain interstitium: implications for Alzheimer disease. J. Biol. Chem. 287, 28163–8 (2012). 22718756 10.1074/jbc.C112.360800 PMC3431649

[b57] Ramos-Fernández E. . Posttranslational nitro-glycative modifications of albumin in Alzheimer’s disease: implications in cytotoxicity and amyloid-β peptide aggregation. J. Alzheimers. Dis. 40, 643–57 (2014). 24503620 10.3233/JAD-130914

[b58] Ishigami A. . Mass spectrometric identification of citrullination sites and immunohistochemical detection of citrullinated glial fibrillary acidic protein in Alzheimer’s disease brains. J. Neurosci. Res. 93, 1664–74 (2015). 26190193 10.1002/jnr.23620

[b59] Kamphuis W. . Glial fibrillary acidic protein isoform expression in plaque related astrogliosis in Alzheimer’s disease. Neurobiol. Aging 35, 492–510 (2014). 24269023 10.1016/j.neurobiolaging.2013.09.035

[b60] Ghoneim F. M. . Protective effect of chronic caffeine intake on gene expression of brain derived neurotrophic factor signaling and the immunoreactivity of glial fibrillary acidic protein and Ki-67 in Alzheimer’s disease. Int. J. Clin. Exp. Pathol. 8, 7710–28 (2015). 26339337 PMC4555665

[b61] Lovell M. A., Xie C. & Markesbery W. R. Decreased glutathione transferase activity in brain and ventricular fluid in Alzheimer’s disease. Neurology 51, 1562–6 (1998). 9855502 10.1212/wnl.51.6.1562

[b62] Ansari M. A. & Scheff S. W. Oxidative stress in the progression of Alzheimer disease in the frontal cortex. J. Neuropathol. Exp. Neurol. 69, 155–67 (2010). 20084018 10.1097/NEN.0b013e3181cb5af4 PMC2826839

[b63] Faux N. G. . An anemia of Alzheimer’s disease. Mol. Psychiatry 19, 1227–34 (2014). 24419041 10.1038/mp.2013.178

[b64] Chuang J.-Y. . Interactions between amyloid-β and hemoglobin: implications for amyloid plaque formation in Alzheimer’s disease. PLoS One 7, e33120 (2012). 22412990 10.1371/journal.pone.0033120 PMC3295782

