# Corrigendum: Macrophage Migration Inhibitory Factor is subjected to glucose modification and oxidation in Alzheimer’s Disease

**DOI:** 10.1038/srep45417

**Published:** 2017-04-05

**Authors:** Omar Kassaar, Marta Pereira Morais, Suying Xu, Emily L. Adam, Rosemary C. Chamberlain, Bryony Jenkins, Tony D. James, Paul T. Francis, Stephen Ward, Robert J. Williams, Jean van den Elsen

Scientific Reports 7: Article number: 4287410.1038/srep42874; published online: 02 23 2017; updated: 04 05 2017

The original version of this Article contained a typographical error in the spelling of the author Tony D. James, which was incorrectly given as Tony James. This has now been corrected in the PDF and HTML versions of the Article.

