# Supplementary material for: Macrophage Migration Inhibitory Factor is subjected to glucose modification and oxidation in Alzheimer’s Disease
Source: Sci Rep. 2017 Feb 23;7:42874. doi: 10.1038/srep42874 (PMC5322340; doi:10.1038/srep42874)
Supplement: Supplementary Information [file srep42874-s1.pdf]

Supplementary Information for:

Macrophage Migration Inhibitory Factor is subjected to glucose modification and oxidation in Alzheimer's Disease

Omar Kassaar[1], Marta Pereira Morais[1], Suying Xu[1], Emily Adam[1], Rosemary Chamberlain[1], Bryony Jenkins[1], Tony James[2], Paul T. Francis[3], Stephen Ward[4], Robert J. Williams[1] and Jean van den Elsen[1]

[1] Department of Biology and Biochemistry, University of Bath, Bath, BA2 7AY, U.K.

[2] Department of Chemistry, University of Bath, Bath, BA2 7AY, U.K.

[3] Institute of Psychiatry, Psychology & Neuroscience, Wolfson Centre for Age Related Diseases, King's College London

[4] Department of Pharmacy and Pharmacology, University of Bath, Bath, BA2 7AY, U.K.

Address correspondence to: Robert Williams (r.j.williams@bath.ac.uk) or Jean van den Elsen (j.m.h.v.elsen@bath.ac.uk)

This PDF file includes:

Supplementary Figures S1 – S11
Supplementary Table 1

Contents of Supplementary Information, Kassaar *et al.*,:

Glucose modification and oxidation of Macrophage Migration Inhibitory Factor in Alzheimer's Disease

Supplementary Figures

**Figure S1**	**A) Anti-AGE western blot of brain tissue lysates**<br>**B) Anti-CML western blot of brain tissue lysates.**
**Figure S2**	**FluPAGE analysis of control MIF incubations**
**Figure S3**	**Mass spectrometry analysis of recombinant MIF**
**Figure S4**	**Protein sequence of recombinant MIF**
**Figure S5**	**Enzyme assays**
**Figure S6**	**A) Glycation protein signature in AD brain**<br>**B) Anti-tau western blots of brain tissue lysates.**
**Figure S7-S11**	**Uncropped images of gels and blots**

Supplementary Tables

Table 1 **Autopsy data of the brain homogenates used in this study**

Supplementary Figures:

Figure S1

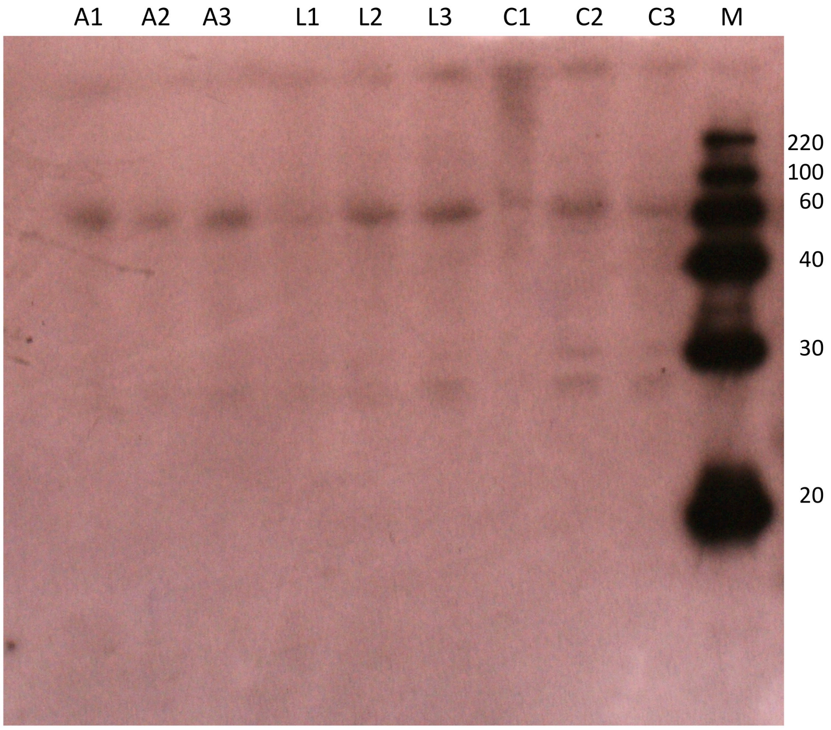

A) Anti-AGE western blot of brain tissue lysates. Homogenates from the temporal cortex of severe (Braak stages V-VI) AD (A1, A2 and A3), mild/moderate (Braak stages I-III) AD (L1, L2 and L3) compared with age matched controls (C1, C2 and C3). The sizes of the marker proteins (M) are given (M_r/1000). AGE-modified proteins can be observed between 25kDa and 60kDa, however no significant differences in AGE signatures are seen between AD and control brains, corresponding to previous findings (Pamplona et al., 2005, Journal of Biological Chemistry 280, p21522). No AGE-modified MIF can be observed in any of the analysed brain lysate samples.

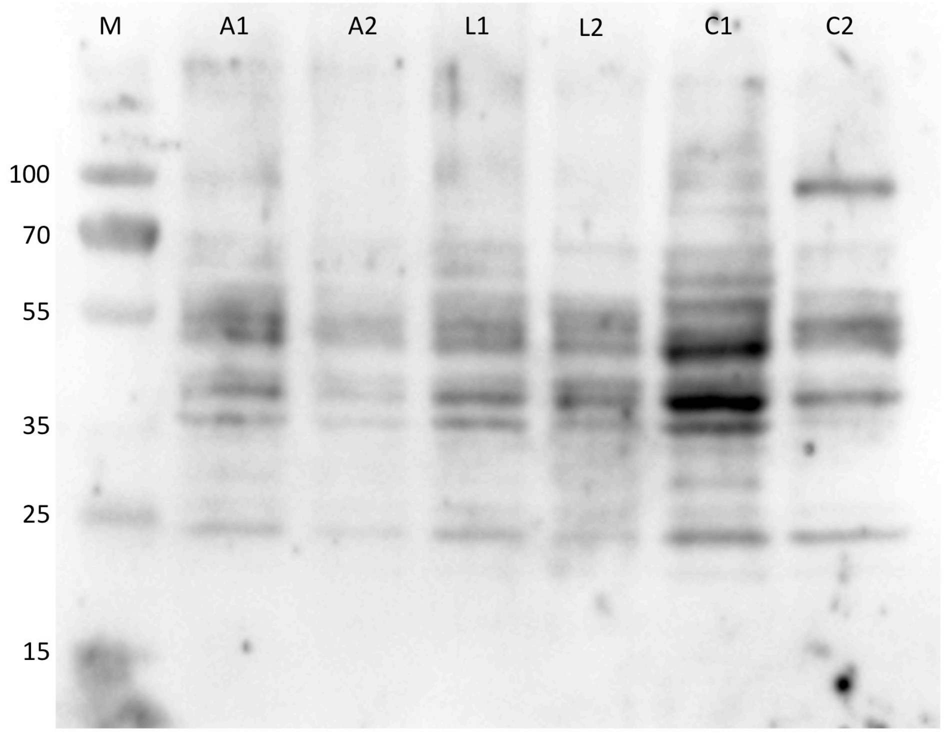

B) Anti-CML western blot of brain tissue lysates. Homogenates from the temporal cortex of severe AD (A1 and A2), mild/moderate AD (L1 and L2) compared with age matched controls (C1 and C2). The sizes of the marker proteins are given (M_r/1000). Similar to the anti-AGE western blot show in S1A, several CML-modified proteins can be observed between 25kDa and 60kDa, however no significant AD-specific CML signatures could be observed in the analysed samples. No CML-modified MIF can be observed in any of the analysed brain lysate samples.

Procedure:

SDS-PAGE

4ul of labelled sample added to 4ul of 2x reducing sample buffer, heated for 5min and loaded on 15% SDS-PAGE gel and electrophoresed.

Anti-AGE/anti-CML western blot

After soaking in transfer buffer for over 30mins at RT, the left hand side of the gel is then blotted (25V for 15min). Blot is placed in 50ml of 5% milk in TBST at RT for 1.5h, and incubated with anti-AGE antibody (Millipore ab9890) or anti-CML antibody (Abcam ab27684). Primary antibody (1:1,000; 0.2%milk in TBST) applied overnight at 4˚C. blot is then washed 4 times 20min each. Overnight labelled blot in primary ab washed 4 times and susbsequently incubated with secondary Ab (anti-Goat HRP) (1:10,000; 0.25% milk in TBST) applied at RT for 1h. Blot was then washed 5 times 30ml each (1x30min wash followed by 4x 20min washes) before applying Pierce ECL substrate and exposed on film.

Figure S2

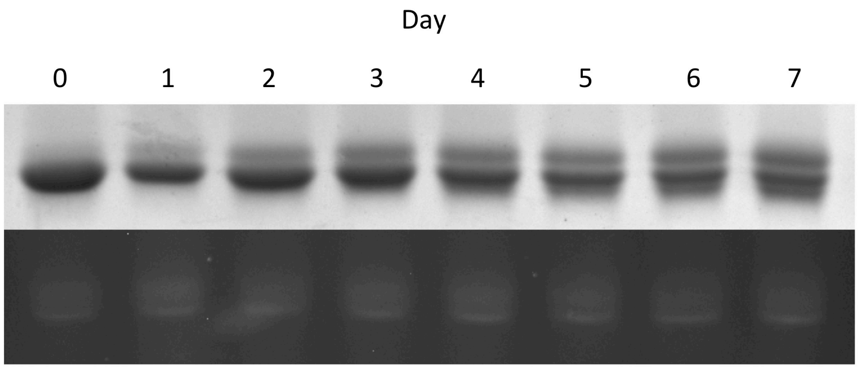

FluPAGE analysis of control MIF incubations. Coomasie stained SDS-PAGE (top) and FluPAGE (bottom) analysis of recombinant MIF after incubation at 37 °C for 7 days. Although an increase of FluPAGE fluorescence was absent in the control experiment, the formation of the doublet can be seen.

Figure S3

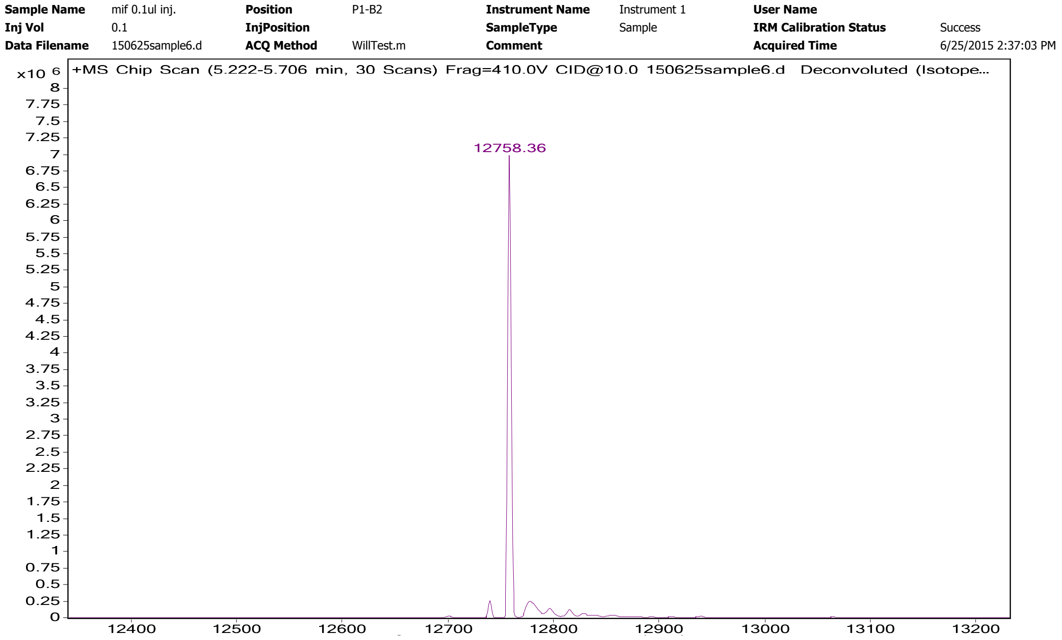

Mass spectrometry analysis of recombinant MIF. MS analysis of recombinant MIF after thrombin cleavage, showing an expected peak with a molecular weight of 12758 Da.

Procedure:
For all of the Mass Spectra used in this report, a micrOTOF ESI-TOF mass spectrometer (Bruker Daltonik GmbH, Bremen Germany) was used. The spectrometer was coupled to an Agilent Technologies 1200 LC system (Agilent Technologies, Santa Clara, CA, USA). To 1mL protein sample (concentration 2 mg/mL) 20 mL of 50:50 water/ACN and 0.2% formic acid was added and 20 mL of this mixture was injected into the mass spectrometer. The nebulizing gas used was nitrogen, which was applied at a pressure of 1 bar. Nitrogen was also used as a drying gas, supplied at a flow rate of 8 L/min and a temperature of 2001C. Positive ion mode was used with a corresponding capillary voltage of 4000V and only full scan data was acquired.

Figure S4

M	G	S	S	H	H	H	H	H	H	S	S	G	L	V	P	R	G	S	H
M	P	M	F	I	V	N	T	N	V	P	*R*	A	S	V	P	D	G	F	L
S	E	L	T	Q	Q	L	A	Q	A	T	G	*K*	P	P	Q	Y	I	A	V
H	V	V	P	D	Q	L	M	A	F	G	G	S	S	E	P	*C*	A	L	*C*
S	L	H	S	I	G	*K*	I	G	G	A	Q	N	*R*	S	Y	S	*K*	L	L
C	G	L	L	A	E	*R*	L	*R*	I	S	P	D	*R*	V	Y	I	N	Y	Y
D	M	N	A	A	N	V	G	W	N	N	S	T	F	A	-				

Protein sequence of recombinant MIF construct
Indicated are the histidine tag (green), thrombin cleavage site (red), the CALC motif (yellow) and the N-terminal proline required for MIF tautomerase activity (blue). Free thiols are in italics. Surface-exposed lysines and arginines, potentially susceptible to glycation, are shown in a larger italicised font (as observed in crystal structures of MIF trimers, e.g. pdb submission code 3L5V).

Figure S5

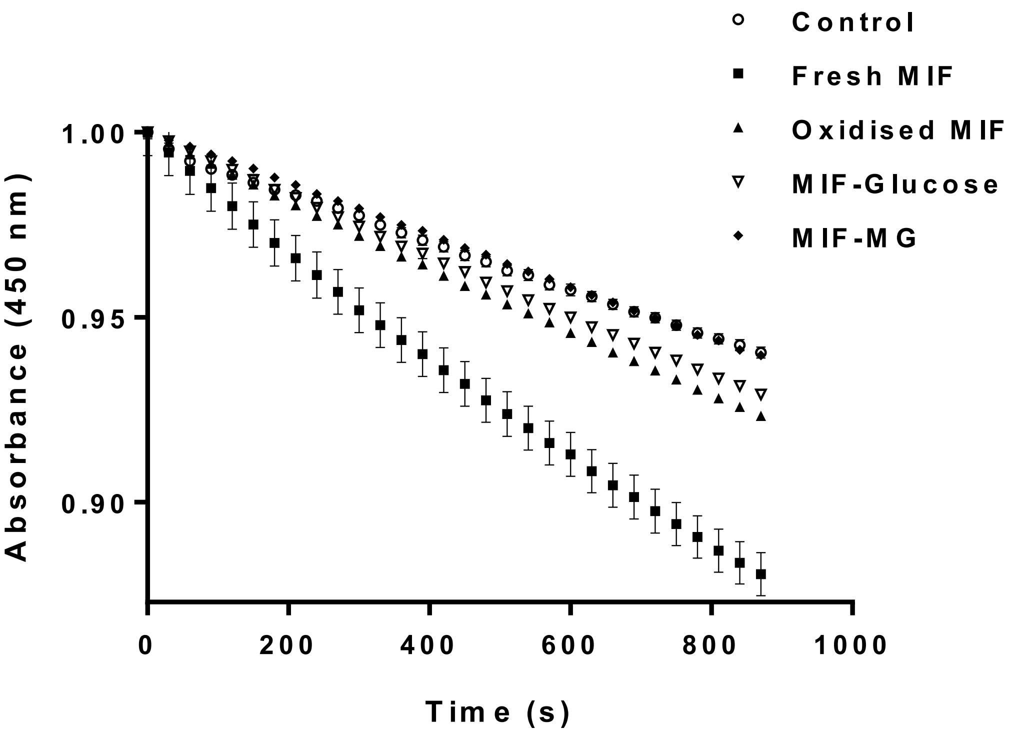

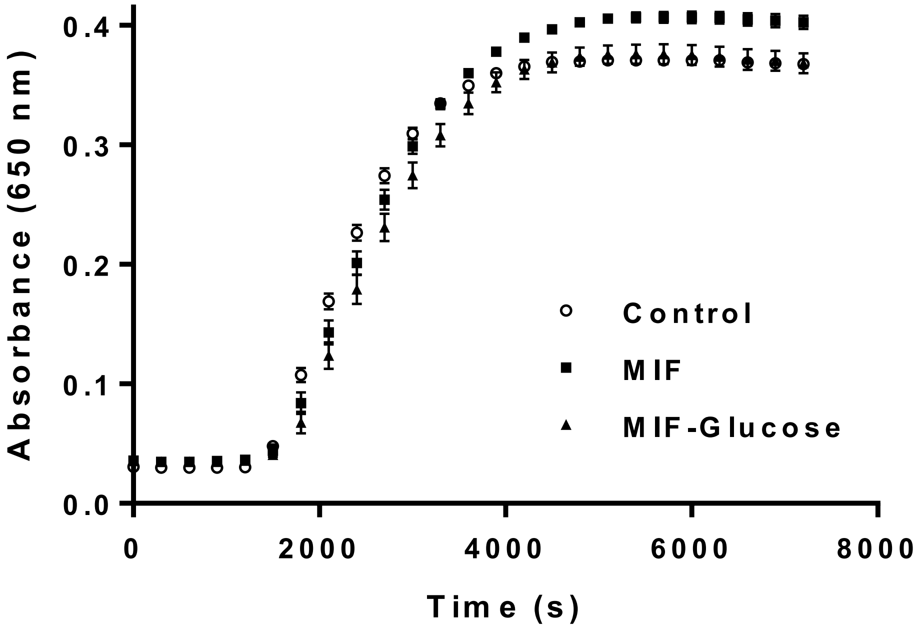

Enzyme assays. MIF tautomerase activity (top) and oxidoreductase activity assay data (bottom). The end point values of these curves were used in Figure 3. Error bars are s.e.m. n>8. See Materials and Methods section for procedures.

Figure S6

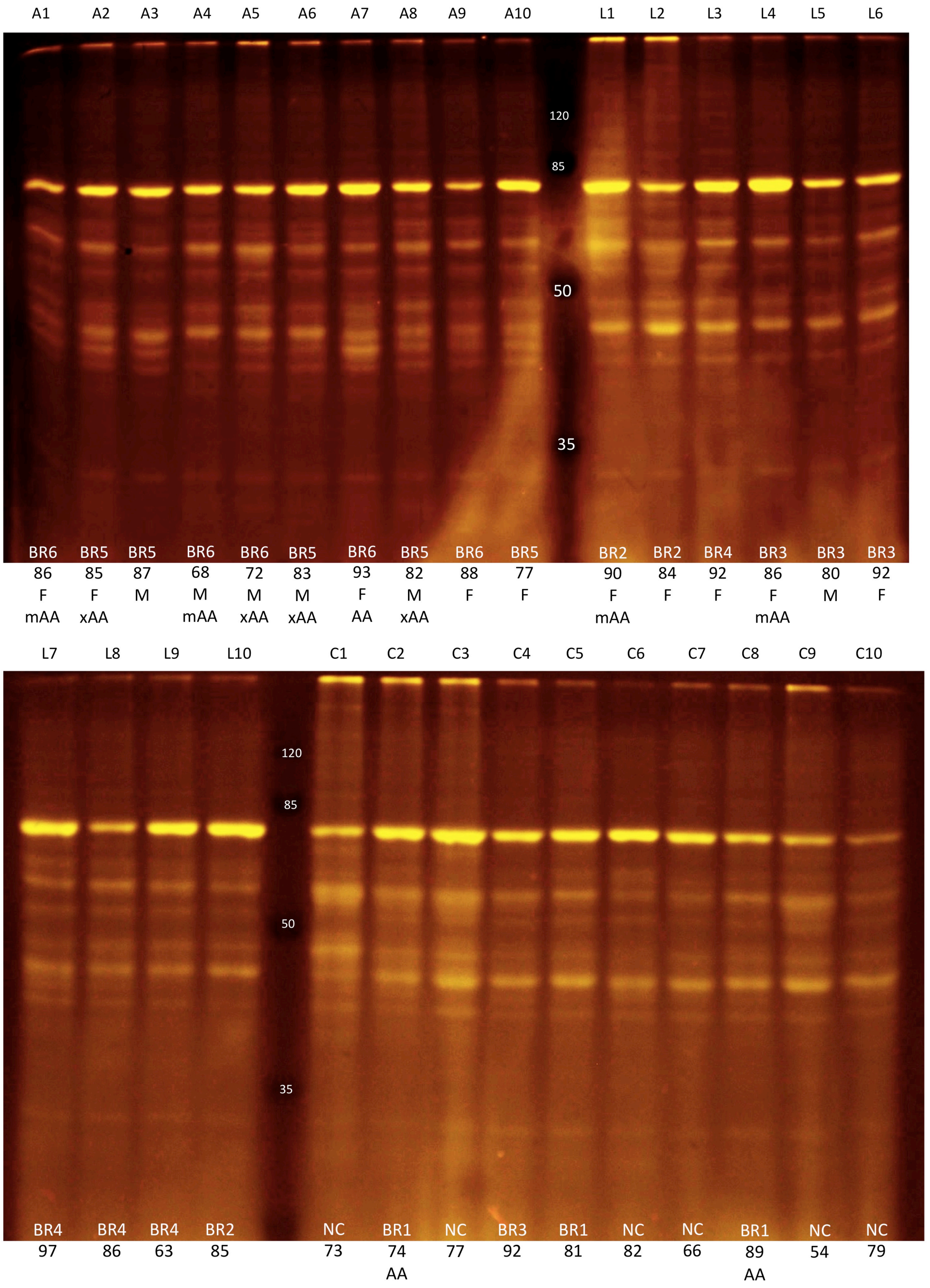

A) Glycation protein signature in AD brain. FluPAGE analysis (12% acrylamide gel) of brain tissue lysates from the temporal cortex of severe/late AD (A1-10, Braak stages V-VI), mild/early AD (L1-10, Braak stage II-IV) compared with age matched controls (C1-10, no Braak classification - Braak stage II). See Supplementary Table 1 for more autopsy data of individual samples. This figure illustrates that in addition to MIF (not included on this molecular weight range), other differential glycation signatures can be observed between Alzheimer’s samples, mild/early AD and age-matched control brains. Apart from HSA (bright band ~70kDa) and GFAP (~45 kDa), many of these proteins are yet to be identified and will be the subject of future investigations.

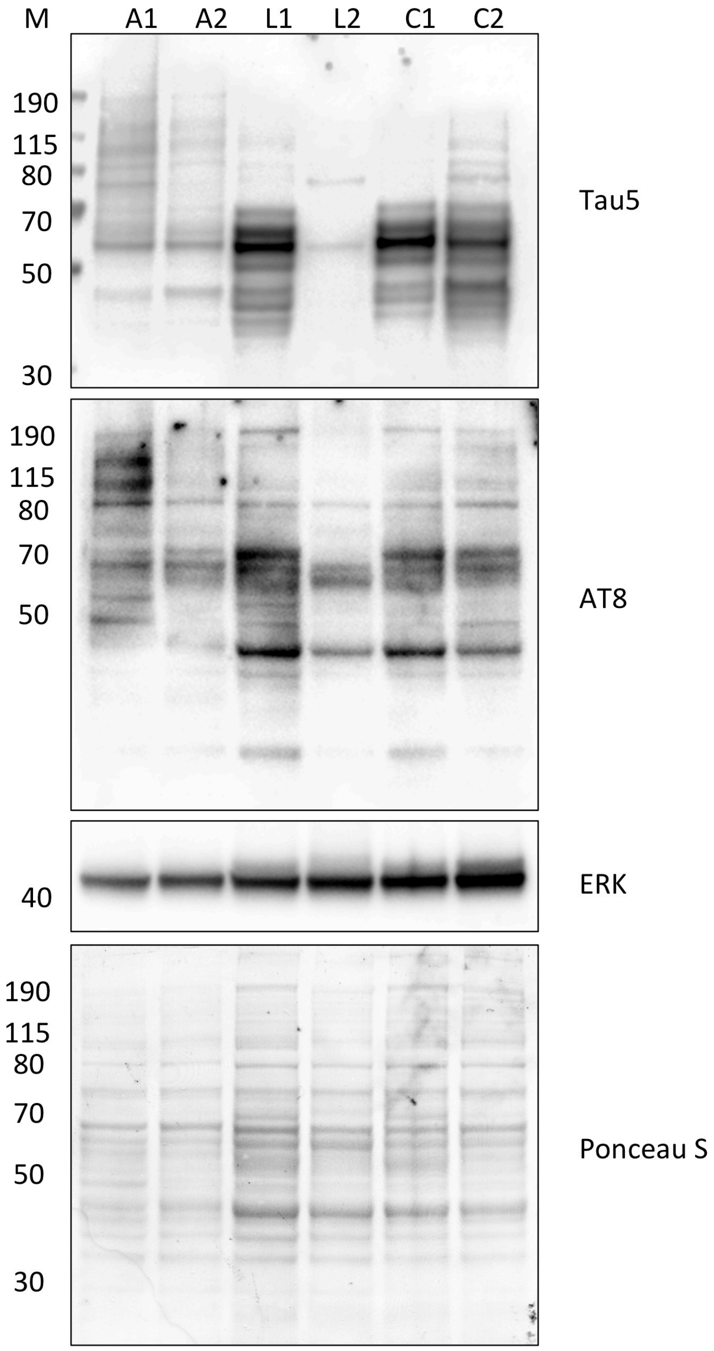

B) Anti-tau and anti-phospho-tau western blots of brain tissue lysates. Homogenates from the temporal cortex of severe AD (A1 and A2), mild/moderate AD (L1 and L2) compared with age matched controls (C1 and C2). We observe the expected total tau profile for AD (top, Tau5), characterised by the high molecular weight protein aggregates that are mostly absent in the L and C samples. In the phospho-tau western blot (middle, AT8) we observed greater phosphorylation of Ser202 and Thr205, which is typically associated with AD. The sizes of the marker proteins are given (M_r/1000). The western blotting procedure is the same as in Supplementary Figure S1 with the exception that anti-tau antibody (Abcam, ab80579) and anti-phospho-tau (ThermoFisher, AT8) were used as primary antibody. Protein levels were assessed by anti-ERK blot and Ponceau S staining.

Figure S7

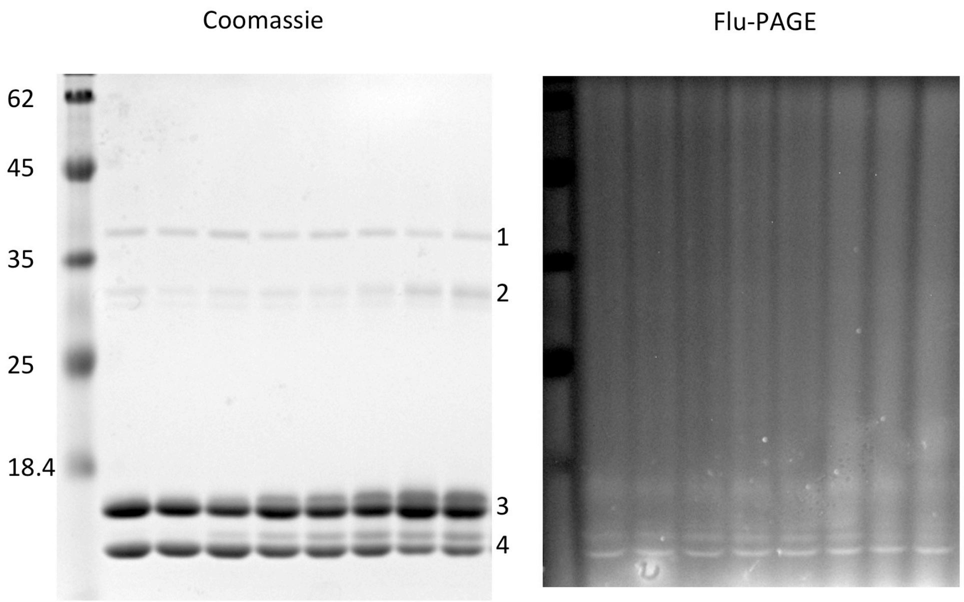

Glycation and oxidation of MIF. Original SDS-PAGE gel of glycated recombinant MIF after glycation with glucose (50 mM) at 37 °C for 7 days, as shown in Figure 2 (a). Shown on the right is the Flu-PAGE analysis of the gel. The coomassie stained version of the same gel is shown on the left. Positions of the molecular weight markers are indicated. Additional bands seen on the coomassie stained gel include: 1) thrombin, 2) recombinant histidine-tagged MIF dimer, 3) recombinant histidine-tagged MIF monomer, 4) thrombin-cleaved MIF. The cropped image in Figure 2 (a) shows the thrombin-cleaved MIF only.

Figure S8

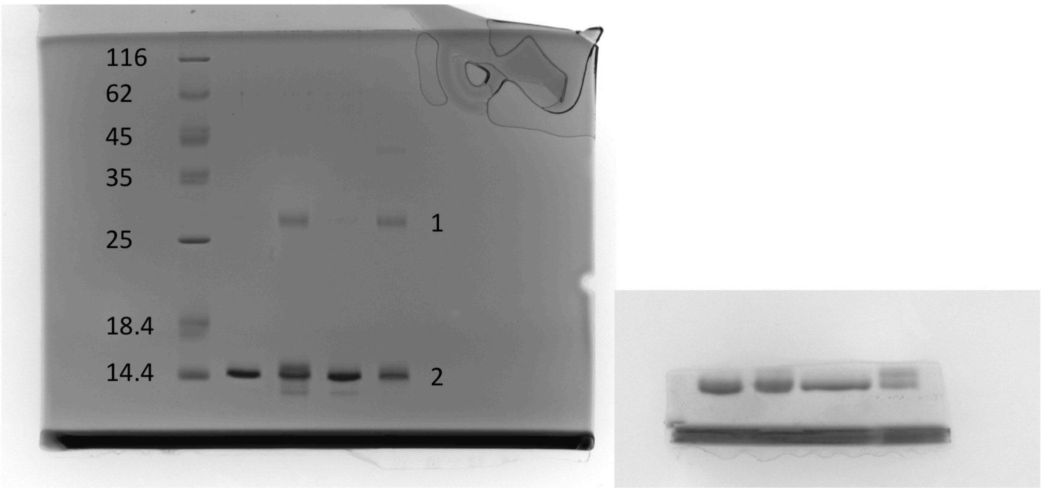

Oxidation of MIF. Original SDS-PAGE gels of MIF oxidation experiment, as shown in Figure 2 (c) and (d). Shown on the left is the coomassie stained SDS-PAGE gel with recombinant MIF incubated in PBS alone, or in the presence of GSH (10 mM) or DTT (5 mM) at 37 °C for 4 days. Positions of the molecular weight markers are indicated. Additional bands seen on the coomassie stained gel include: 1) thrombin-cleaved recombinant MIF dimer, 2) thrombin-cleaved recombinant MIF monomer. The cropped image in Figure 2 (c) shows the thrombin-cleaved MIF only.

Shown on the right is the coomassie stained SDS-PAGE gel with recombinant MIF incubated in the presence of H_2O_2 (1 mM), GSSG (10 mM) or GSNO (400 µM) at 37 °C for 1 hour. This gel ruptured during the coomassie staining process, however the same additional bands could be observed as in the gel on the left, including 1) thrombin-cleaved recombinant MIF dimer, 2) thrombin-cleaved recombinant MIF monomer. The cropped image in Figure 2 (d) shows the thrombin-cleaved MIF only.

Figure S9

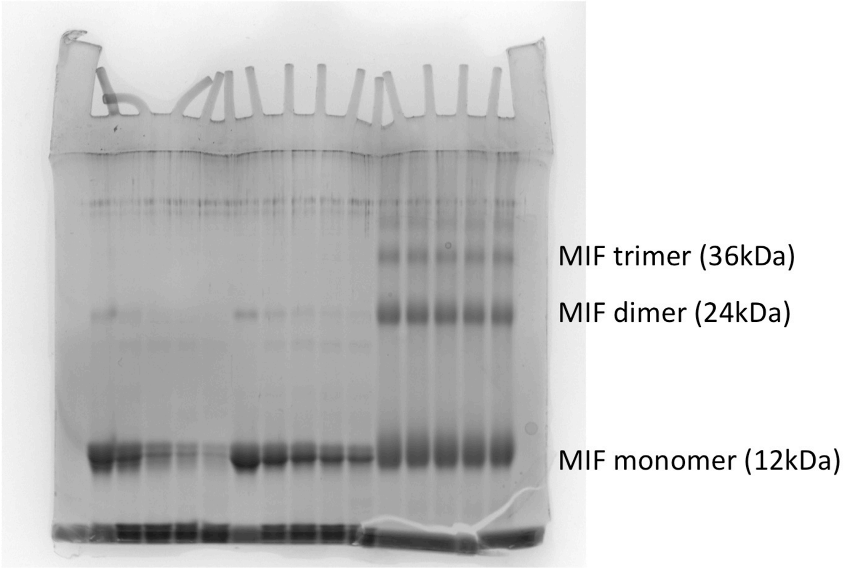

MIF Trypsin digest. Trypsin digest of unmodified (lanes 1-5) and glycated MIF with glucose (lanes 6-10) and MG (lanes 11-15), followed over 4 hours at 37 °C, taking samples at 0h, 1h, 2h, 3h and 4h.

Figure S10

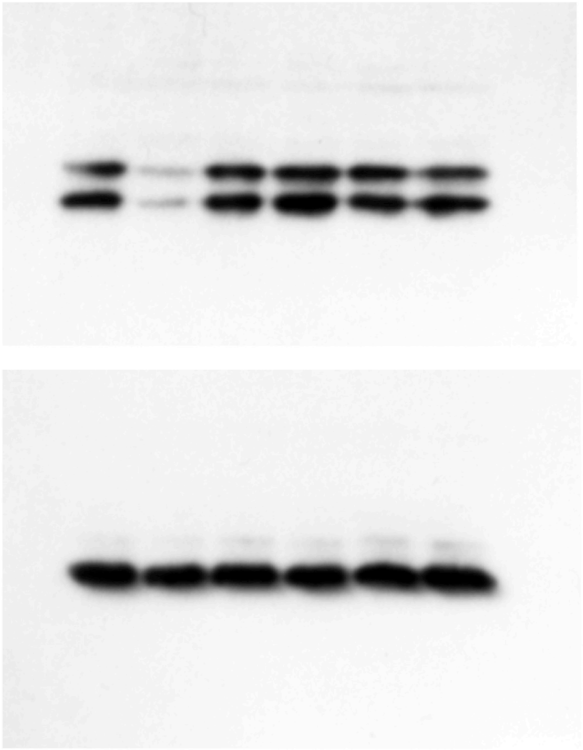

Phosphorylation of ERK. Original Western blot images used for the analysis of the effect of glycated MIF on ERK phosphorylation in primary mouse glial co-cultures of astrocytes and microglia, as shown in Figure 4 (a). Representative blots after 20 hours stained for phosphorylated ERK (top) and total ERK (bottom) assayed by Western blotting, with PBS control, U0126 inhibitor (10 μM), MIF (100 ng/ml and 1,000 ng/ml), glycated MIF (100 ng/ml and 1,000 ng/ml).

Figure S11

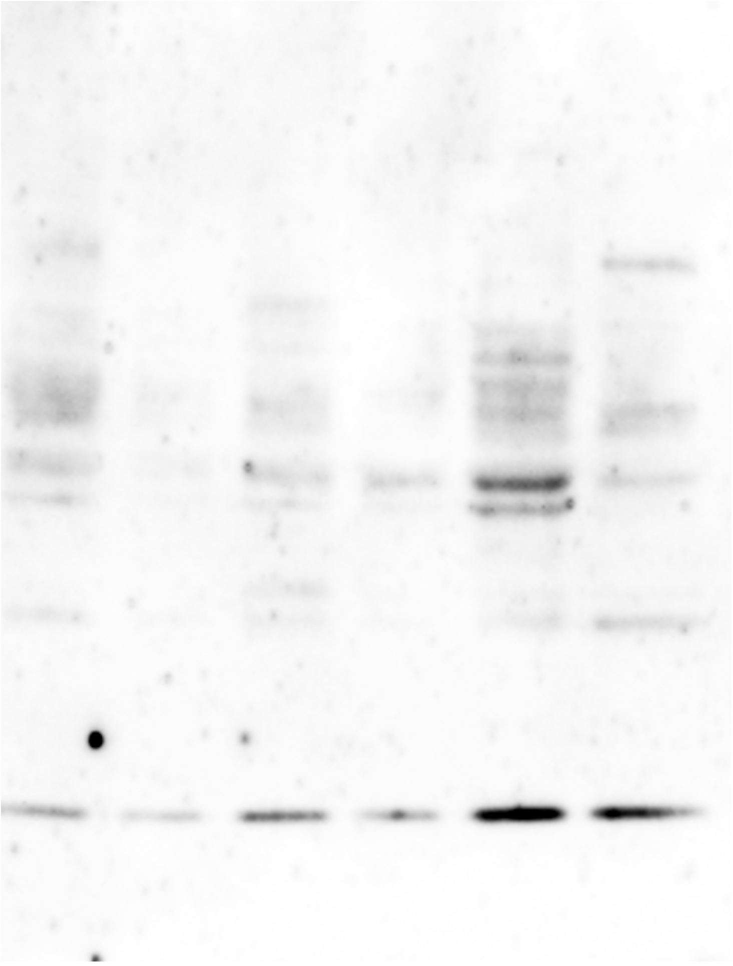

Anti-MIF western blot analysis of brain tissue lysates. Original western blot image used in the analysis of the MIF protein levels in the brain tissues lysates, as shown in Figure 1c. Shown from left-to-right are brain tissue lysates from severe/late AD (lanes 1 and 2), mild/early AD (lanes 3 and 4) and age matched controls (lanes 5 and 6).

Supplementary Table 1

Autopsy data of the brain homogenates used in this study

Sample	Age (Years)	Sex	Post-mortem delay (Days)	Pathological Diagnosis
**Severe/late**	**AD**			
A1	86	F	71	Alzheimer's disease, HP-tau stage VI with mild amyloid angiopathy
A2	85	F	40	Alzheimer's disease, HP-tau Braak stage V with marked amyloid angiopathy
A3	87	M	55	Alzheimer's disease (BNE stage 5)
A4	68	M	25	Alzheimer's disease, HP-tau Braak stage VI with mild to moderate amyloid angiopathy
A5	72	M	5	Alzheimer's disease, Braak VI with marked amyloid angiopathy
A6	83	M	77	Alzheimer's disease, BNE modified Braak stage V with extensive amyloid angiopathy
A7	93	F	38	Alzheimer's disease, HP-tau stage VI with moderate amyloid angiopathy
A8	82	M	34	Alzheimer's disease, (BNE modified Braak stage V) with extensive amyloid angiopathy
A9	88	F	66	Alzheimer's disease, Braak stage VI
A10	77	F	42	Alzheimer's disease (modified Braak BNE stage V), with more predominant posterior pathology & limbic and mild cortical TDP-43 pathology
**Mild/Early**	**AD**			
L1	90	F	50	Control brain, mild alzheimer-type changes (modified Braak stage II) and mild amyloid angiopathy
L2	84	F	53	Alzheimer's changes, Braak II, consistent with patient age
L3	92	F	45	Alzheimer's changes, Braak IV, consistent with patient age
L4	86	F	43	Alzheimer's-type pathology (BNE stage 3)
L5	80	M	60	Alzheimer's disease, Braak stage III with mild amyloid angiopathy
L6	92	F	20	Alzheimer's disease, moderate/limbic stage Braak III
L7	97	F	68	Alzheimer's disease, probable Braak III or IV
L8	91	M	28	Alzheimer's disease (BNE stage 4, CERAD probable); TDP-43 pathology in hippocampal region

Sample	Age (Years)	Sex	PMD (Days)	Pathological Diagnosis
L9	63	M	16	Alzheimer's changes, Braak stage IV consistent with aging
L10	85	M	64	Alzheimer's type changes, Braak II
**Control**				
C1	73	M	23	Normal control
C2	74	M	23	Control brain (AD modified Braak stage I); amyloid angiopathy
C3	77	M	10	Normal control
C4	92	F	30	Control; consistent with ageing (tau Braak stage III)
C5	81	M	18	Control, old cerebral infarct (Braak stage I)
C6	82	F	43	Normal control
C7	66	M	52	Normal control, minimal ageing changes (consistent with HP-tau stage +)
C8	89	F	41	Control case but with Hypoxic-type changes and amyloid angiopathy (AD BNE modified Braak score I-II)
C9	54	M	31	Normal control
C10	79	M	24	Normal control, mild ageing changes
